# Exposure to nature is associated with decreased functional connectivity within the distress network: A resting state EEG study

**DOI:** 10.3389/fpsyg.2023.1171215

**Published:** 2023-04-20

**Authors:** Claudio Imperatori, Chiara Massullo, Elena De Rossi, Giuseppe Alessio Carbone, Annalisa Theodorou, Massimiliano Scopelliti, Luciano Romano, Claudia Del Gatto, Giorgia Allegrini, Giuseppe Carrus, Angelo Panno

**Affiliations:** ^1^Cognitive and Clinical Psychology Laboratory, Department of Human Sciences, European University of Rome, Rome, Italy; ^2^Experimental Psychology Laboratory, Department of Education, Roma Tre University, Rome, Italy; ^3^Department of Psychology, University of Turin, Turin, Italy; ^4^Department of Human Studies, Libera Università Maria SS. Assunta (LUMSA), Rome, Italy

**Keywords:** nature exposure, contact with nature, restorativeness, simulated nature, EEG, functional connectivity, distress network, eLORETA

## Abstract

**Introduction:**

Despite the well-established evidence supporting the restorative potential of nature exposure, the neurophysiological underpinnings of the restorative cognitive/emotional effect of nature are not yet fully understood. The main purpose of the current study was to investigate the association between exposure to nature and electroencephalography (EEG) functional connectivity in the distress network.

**Methods:**

Fifty-three individuals (11 men and 42 women; mean age 21.38 ± 1.54 years) were randomly assigned to two groups: (i) a green group and (ii) a gray group. A slideshow consisting of images depicting natural and urban scenarios were, respectively, presented to the green and the gray group. Before and after the slideshow, 5 min resting state (RS) EEG recordings were performed. The exact low-resolution electromagnetic tomography (eLORETA) software was used to execute all EEG analyses.

**Results:**

Compared to the gray group, the green group showed a significant increase in positive emotions (*F*_1; 50_ = 9.50 *p* = 0.003) and in the subjective experience of being full of energy and alive (*F*_1; 50_ = 4.72 *p* = 0.035). Furthermore, as compared to urban pictures, the exposure to natural images was associated with a decrease of delta functional connectivity in the distress network, specifically between the left insula and left subgenual anterior cingulate cortex (*T* = −3.70, *p* = 0.023).

**Discussion:**

Our results would seem to be in accordance with previous neurophysiological studies suggesting that experiencing natural environments is associated with brain functional dynamics linked to emotional restorative processes.

## Introduction

The literature on contact with nature has consistently shown that mere exposure to certain types of natural environments may cause beneficial effects on humans (e.g., Bowler et al., 2010; Hartig et al., 2014; Houlden et al., 2018; Beute et al., 2020; Gladwin et al., 2022). One of the most important changes that have been observed in people when they are exposed to nature is psychological restoration and stress reduction (Yao et al., 2021). In this regard, two prominent theories have been used to explain these effects. First, the Stress Reduction Theory (SRT; Ulrich, 1993) postulates that nature provokes automatic psychophysiological responses that modulate stress. The authors support the idea that living in urban areas may cause stress of which we are not fully aware, and, accordingly, nature can reduce it outside the consciousness. Second, the Attention Restoration Theory (ART; Kaplan and Kaplan, 1989) that sustains how nature restores voluntary attention, namely a type of attention requiring active effort for the individual to focus on an object meanwhile suppressing all the distracting stimuli. This occurs because the natural environment stimulates a type of involuntary and effortless attention, allowing for our cognitive functions to restore. Both the SRT and the ART see the changes observed in individuals as evolutionary responses to environments that can guarantee high rates of survival (Bratman et al., 2012). In this regard, Kaplan (1995) has convincingly discussed the potential integration of the two theories and related processes. Interestingly, these restorative effects have been proved to be comparable, although not completely overlapping, both in natural (outdoors) and simulated (indoors) natural environments, including virtual ones (Kjellgren and Buhrkall, 2010; Bratman et al., 2012; Pasca et al., 2022; Spano et al., 2022; Theodorou et al., 2023a,b).

Despite the well-established evidence supporting the restorative potential of nature exposure, the neurophysiological underpinnings of the restorative cognitive/emotional effect of nature are not yet fully understood. Two recent systematic reviews and meta-analyses that focused on both self-reported measures and psychophysiological bio-signals (i.e., systolic and diastolic blood pressure, heart rate variability, and cortisol levels) showed that exposure to natural environments is associated with stress reduction and/or enhanced relaxation (Mygind et al., 2021; Yao et al., 2021).

Similar findings have been also reported in studies investigating brain functional dynamics by means of different neuroimaging techniques (Norwood et al., 2019). In the same way, it has been detected that the exposure to urban stimuli is related with the activation of brain areas and/or large-scale brain networks linked to stress and negative affect (Ancora et al., 2022).

Particularly, the electroencephalography (EEG) is widely used for the investigation of brain activity related to the exposure of natural settings (Grassini et al., 2022), providing an important source of information on changes in neurophysiological dynamics associated with stress-related processes (Alonso et al., 2015; Giannakakis et al., 2019; Saeed et al., 2020; Massullo et al., 2022). More specifically, compared to the traditional visual EEG scoring, through quantitative EEG (qEEG; i.e., numerical computations of parameters from the EEG data) it is possible to estimate different parameters, such as EEG power spectra (i.e., the power distribution of EEG series in the frequency domain), that offer a significant source of information in terms of brain activity (Thatcher, 2010; Lehembre et al., 2012).

In this research field, previous EEG studies showed that experiencing natural environments is associated with several neurophysiological patterns linked to stress reduction, including increased alpha (Ulrich, 1981; Chang et al., 2008; Grassini et al., 2019, 2022; Sahni and Kumar, 2020; Koivisto et al., 2022; Olszewska-Guizzo et al., 2022b) and theta power (Olszewska-Guizzo et al., 2022b), as well as decreased beta activity (Grassini et al., 2019).

Of relevance, an important advance in the analysis of EEG signal is the investigation of functional connectivity referring to the calculation of *“various measures of neural dynamics to functional brain state, determined by behavior, cognition, or neuropathology”* (Srinivasan et al., 2007). Particularly, EEG connectivity-based measures, such as EEG coherence, provide a valuable estimation of the functional interactions between brain structures operating in specific frequency bands, offering significant information about network dynamics and functional integration across neural systems (Srinivasan et al., 2007; Whitton et al., 2018).

To the best of our knowledge, only one study has investigated the association between EEG functional connectivity and exposure to nature. Chen et al. (2020) showed that, as compared to a nonrestorative scenario (i.e., a traffic island), a restorative one (i.e., a wooded garden) was associated with a significant increase of theta connectivity. Therefore, in order to extend previous EEG findings, the main aim of the current study was to investigate the association between exposure to nature and EEG functional connectivity among brain nodes involved in emotional distress (i.e., the distress network; De Ridder et al., 2011; Pattyn et al., 2018). According to the view that natural environments can lead to stress reduction (Norwood et al., 2019; Mygind et al., 2021; Yao et al., 2021), we hypothesized that compared to images depicting urban spaces (i.e., gray condition), pictures showing green spaces (i.e., green condition) would be associated with a decrease of functional connectivity within the distress network.

## Materials and methods

### Participants

An *a priori* power analysis was conducted using G*Power 3.1 software (Faul et al., 2009). It revealed that, given a probability level of 0.05, a sample size of 52 was required to provide a satisfactory statistical power (1–β = 80%) with large effect size (i.e., *d* = 0.80) in a two-sided test. Given the novelty of the study, this effect size was estimated according to a previous EEG connectivity report in this research field (Chen et al., 2020).

Participants were recruited in the campus of the European University of Rome through advertising. The enrollment lasted from November 2021 to May 2022. The following inclusion criteria were applied: right-handedness [i.e., Laterality Quotient > of 60 according to the Edinburgh Handedness Inventory–short form (EHI–SF; Veale, 2014)], self-reported lifetime negative anamnesis of neurological diagnosis, normal or corrected-to-normal vision, absence of use of active drugs (including psychotropic medications) on the central nervous system in the 2 weeks before the study. A checklist with dichotomous items was administered to assess inclusion criteria as well as socio-demographic data (e.g., age, educational attainment) and clinical data (e.g., tobacco and alcohol use in the last 6 months).

Eighty-seven university students were assessed for eligibility after providing their informed consent. Fifty-eight participants fulfilled the inclusion criteria and were enrolled in the present research. Five participants (8.62%) had no suitable EEG recordings (i.e., bad EEG signal), thus we have excluded such participants from the analyses. Therefore, the final sample consisted of 53 participants (11 men and 42 women; mean age 21.38 ± 1.54 years). Participants received course credits for their participation and were not aware about the experimental hypothesis. This research was approved by the European University’s ethics review board (Prot. N. 11/2021) and was performed according to the Helsinki declaration standards.

### Study design and procedures

In line with previous experimental research on exposure to natural and built environments (e.g., Chen et al., 2020), the current study was performed in the following three consecutive time-points.

#### Pre-stimuli assessment (T0)

After providing the written informed consent, all participants underwent a 5 min resting state (RS) EEG recording (T0-RS-EEG condition) and were administered the state version of the Positive and Negative Affect Schedule (PANAS; Watson et al., 1988), and the state version of the Subjective Vitality Scale (SVS-S; Ryan and Frederick, 1997).

#### Stimuli administration

After the pre-assessment phase, participants were randomly assigned to the two groups using the online software Research Randomizer:^1^ (i) the green group and (ii) the gray group. A slideshow consisting of images depicting a natural and an urban scenarios were, respectively, presented to the green and the gray group. More specifically, 20 natural and urban digitized color pictures were used. Images were presented for 12 s and were preceded by a fixation cross placed centrally on the screen with a duration of 1 s. Participants were instructed to freely explore each picture.

#### Post-stimuli assessment (T1)

Immediately at the end of the slideshow, all participants underwent another 5 min RS EEG recording (T1-RS-EEG condition) and were asked to complete again the state version of the PANAS and the SVS-S. All participants also completed the Brief Symptoms Inventory (BSI; Derogatis and Melisaratos, 1983) and were asked to evaluate the pleasantness (i.e., *“To what extent did you like the images shown?*”), the quality (i.e., “*How would you rate the quality of the images shown?*”), the lightness (i.e., “*How would you rate the lightness of the images shown?*”), and the familiarity (i.e., “*How familiar are you with the place you saw in the pictures?*”) of the stimuli using a 10-point visual analog scale. These measures have been frequently used in earlier research on exposure to natural and built environments (e.g., Carrus et al., 2013).

### EEG recordings and analysis

All EEG recordings were performed in an eye-closed RS condition with participants sitting on a comfortable chair (Massullo et al., 2020). EEG data were recorded using a 31-electrode cap placed according to the International 10–20 System and acquired using Micromed System Plus digital EEGraph (Micromed© S.p.A., Mogliano Veneto, TV, Italy). In order to avoid the possible effect of alcohol and caffeine on EEG data, participants were asked to refrain from them immediately before their EEG recordings (i.e., at least 4 h). All EEG recordings lasted 5 min and were carried out with the following parameters: 256 Hz sampling rate, and A/D conversion performed at 16 bits. The reference electrodes were placed on the right mastoid. Impedances were kept below 5 kΩ before starting EEG recording and checked again at the end of EEG session (i.e., T0 and T1) for each participant.

EEG recordings were offline filtered 1–40 Hz (with values corresponding, respectively, to high and low frequency band-pass filters) using the EEGLAB toolbox for MatLab v.2022.1 (Delorme and Makeig, 2004). Successively, the average reference was computed and a first visual inspection of the most noticeable and severe artifacts was carried out. Furthermore, in order to remove the main electrical, muscular, and visual artifacts, an independent component analysis (ICA) based on the infomax decomposition algorithm (“runica” tool of EEGLAB), was applied to all EEG channels. After the ICA decomposition, bad components were removed using the MARA EEGLAB plug-in and by visual inspection (Winkler et al., 2011, 2014). Subsequently, the identification of artifact-contaminated channels was mainly carried out in two ways: firstsly, the data spectrum of the channels was observed using the Plot → Channel spectra and maps tool; secondly, the EEGLAB’s “automatic channel rejection” function was further used to identify channels with kurtosis above five z-scores. Finally, a three-dimensional spherical spline interpolation was performed on the most artifact-contaminated channels (Perrin et al., 1989; Ferree, 2006).

The minimum length of the artifact-free EEG recording included in the analysis was 200 s (even if not consecutive) for each participant for each condition. Artifact-free data were then fragmented into epochs of 4 s for the EEG coherence analysis (Miljevic et al., 2022).

The exact Low-Resolution Brain Electromagnetic Tomography software (eLORETA; Pascual-Marqui et al., 2011) was used for all EEG analyses (including epochs fragmentation). This is a validated computer program for the investigation of large-scale brain networks functional connectivity (Liu et al., 2018). The eLORETA is considered one of the most widely adopted systems among the brain source localization procedures (Jatoi et al., 2014; Halder et al., 2019; Asadzadeh et al., 2020) able to detect electrocortical activity with rigorous localization capacity in terms of cortical, limbic (e.g., the hippocampus and the amygdaloid complex) and para-limbic (e.g., the insula) structures, even when reduced electrode montages (i.e., <30) have been applied (e.g., Mulert et al., 2004; Zotev and Bodurka, 2020).

In the current study, in order to evaluate the connectivity in the distress network, eight Regions of Interest (ROIs; Table 1) were pre-defined according to a previous LORETA study (Pattyn et al., 2018) choosing the “ROI-maker#2 method” (i.e., based on the Brodmann’s areas) available in the eLORETA software.

**Table 1:** eLORETA Montreal Neurological Institute (MNI) coordinates of the distress network.

Regions of interest	Brodmann areas	eLORETA MNI coordinates^*^		
		*x*	*y*	*z*
Left insula	13	−40	−10	10
Right insula	13	40	−5	10
Left amygdala	34 + 35	−20	−10	−20
Right amygdala	34 + 35	20	−10	−20
Left sgACC	25	−10	20	−15
Right sgACC	25	5	15	−15
Left dACC	24 + 32	−10	15	30
Right dACC	24 + 32	5	15	30

MNI, montreal neurological institute; eLORETA, exact low-resolution brain electromagnetic tomography software; sgACC, subgenual anterior cingulate cortex; and dACC, dorsal anterior cingulate cortex.

*eLORETA ROIs coordinates reconstruction.

The connectivity analysis was performed by computing the Lagged Phase Synchronization (LPS; Pascual-Marqui et al., 2011), one of the main neurophysiological indices used for the assessment of brain functional connectivity (Olbrich et al., 2014; Hata et al., 2016; Imperatori et al., 2017). The LPS reduces the artifacts (e.g., the volume-conduction) by removing the instantaneous zero-lag contribution (Hata et al., 2016), and it calculates the similarity between signals in the frequency domain, based on normalized Fourier transform (performed through the eLORETA), with values ranging from 0 (i.e., no synchronization) to 1 (i.e., the maximum synchronization).

According to previous studies (e.g., Canuet et al., 2011; de la Salle et al., 2016; Kitaura et al., 2017), in order to perform the source reconstruction of the brain networks, the “single nearest voxel” option (i.e., each ROI consisting of a single voxel, the closest to each seed) was selected in the eLORETA software. In the current study, the following four frequency bands were considered (Mazza et al., 2014): delta (0.5–4 Hz), theta (4.5–7.5 Hz), alpha (8–13 Hz), and beta (13.5–30 Hz).

### Self-report questionnaires

The PANAS (Watson et al., 1988) is a 20-item self-report questionnaire assessing both negative (e.g., scared, distressed) and positive affect (e.g., strong, enthusiastic). In the current study, the “state” (i.e., “*at this moment*”) Italian version of the PANAS (Terraciano et al., 2003) was used. At T0, the Cronbach’s alpha values were 0.81 for the positive and 0.86 for the negative subscale. At T1, the Cronbach’s alpha values were 0.90 for both positive and negative subscale.

The SVS-S (Ryan and Frederick, 1997) is a seven-item self-report questionnaire assessing subjective experience of being full of energy and alive. Each item is rated on a seven-point Likert scale ranging from “not at all” to “very true,” with higher scores indicating greater subjective vitality. In the current study, the Italian version of the SVS-S was used, and the Cronbach’s alpha values were 0.81 and 0.86 for T0 and T1, respectively.

The BSI (Derogatis and Melisaratos, 1983) is a self-report questionnaire widely used to assess psychological symptoms (e.g., anxiety and depressive symptoms). It is composed of 53 items rated on a five-point Likert scale (0–4), with higher scores indicating more severe self-reported symptoms. The BSI provides a Global Severity Index (GSI), which is designed to measure overall psychopathological distress. This questionnaire is characterized by good internal consistency and test–retest reliability (Derogatis and Melisaratos, 1983). In the current study, the Italian version of the scale (Adawi et al., 2019) was used and the Cronbach’s alpha was 0.96 for the GSI.

### Statistical analysis

In order to investigate the association between nature exposure and EEG functional connectivity in the distress network, the following comparisons were performed: (i) T0-RS-EEG green group vs. T0-RS-EEG gray group, and (ii) T1-RS-EEG green group vs. T1-RS-EEG gray group. Comparisons were performed using the statistical non-parametric mapping methodology provided by the eLORETA software (Nichols and Holmes, 2002). This approach is based on Fisher’s permutation test and used a nonparametric randomization procedure in order to perform the correction of significance for multiple testing. Briefly (for more details see Kitaura et al., 2017; Hata et al., 2019), this procedure computes 5,000 data randomizations to determine the critical probability threshold of *T*-values corresponding to a statistically corrected (i.e., after the multiple comparisons among each ROIs in each frequencies) *p* values (*p* = 0.05 and *p* = 0.01). The eLORETA software also computed the effect size thresholds for *T*-statistics corresponding to Cohen’s *d* values (Cohen, 1988): small = 0.2, medium = 0.5, and large = 0.8.

Two-way chi-squared and univariate ANOVAs were used to analyze differences between groups at T0, respectively for dichotomous and dimensional measures. According to the recommendations for pre-post study designs (Senn, 2006), questionnaire data were analyzed using analysis of covariance (ANCOVA) with group (green vs. gray) as a between-subject factor, value at T1 as dependent variables, and value at T0 as a covariate. In order to determine effect sizes, Cohen’s d and Cohen’s *d_ppc2_* (Morris, 2008; equation number 8) values were calculated for ANOVAs and ANCOVA analyses, respectively. Chi-squared, ANOVA and ANCOVA analyses were performed using SPSS (version 18.0).

## Results

For all study participants the qualitative visual evaluation of the EEG recordings (i.e., T0-RS-EEG and T1-RS-EEG) showed no relevant evidence of unusual neurophysiological patterns, such as epileptic discharges and/or focal delta slow waves. In the T0-RS-EEG condition the average epochs analyzed for the green group and the gray group was 70.54 ± 6.62 and 68.56 ± 9.28, respectively (*F*_1; 51_ = 0.797 *p* = 0.376). In the T1-RS-EEG condition, the average epochs analyzed for green group and gray group was 70.88 ± 8.16 and 66.96 ± 7.17, respectively (*F*_1; 51_ = 3.466, *p* = 0.068).

The two groups did not significantly differ in age and in other socio-demographic variables. At T1, a significant group main effect was observed for PANAS positive dimension and SVS-S total score. Specifically, compared to the gray group, the green group showed a significant increase of positive emotions (green group: 28.62 ± 8.70 vs. gray group: 24.70 ± 7.41; *F*_1; 50_ = 9.50; *p* = 0.003) and SVS-S total score (green group: 27.42 ± 8.05 vs. gray group: 23.15 ± 7.48; *F*_1; 50_ = 4.72; *p* = 0.035). Detailed descriptive and F statistics are reported in Table 2.

**Table 2:** Detailed descriptive and F statistics.

Variable	Green group (*N* = 26)	Gray group (*N* = 27)	Test statistics	*p*	Effect size
Age: M ± DS	21.15 ± 1.43	21.59 ± 1.62	F_1;51_ = 1.084	0.303	
Women: *N* (%)	21 (80.8%)	21 (77.8%)	χ^2^ = 0.072	0.788	
Tobacco use: *N* (%)	12 (46.2%)	10 (37%)	χ^2^ = 0.453	0.501	
Alcohol use: *N* (%)	23 (88.5%)	24 (.9%)	χ^2^ = 0.002	0.961	
BSI-GSI: M ± DS	0.85 ± 0.68	0.89 ± 0.75	F_1;51_ = 0.047	0.829	*d =* 0.056
Stimuli pleasantness: M ± DS	7.46 ± 1.45	3.33 ± 1.47	F_1;51_ = 106.14	**< 0.001**	*d =* −2.828
Stimuli quality: M ± DS	7.19 ± 1.86	5.04 ± 1.99	F_1;51_ = 16.602	**< 0.001**	*d =* −1.116
Stimuli lightness: M ± DS	7.69 ± 1.78	5.37 ± 2.20	F_1;51_ = 17.693	**< 0.001**	*d =* −1.157
Stimuli familiarity: M ± DS	8.19 ± 1.44	5.85 ± 2.49	F_1;51_ = 17.336	**< 0.001**	*d =* −1.145
PANAS-N T0: M ± DS	18.00 ± 5.38	15.19 ± 5.95	F_1;51_ = 3.255	0.077	*d =* −0.495
PANAS-P T0: M ± DS	29.15 ± 6.82	30.15 ± 5.13	F_1;51_ = 0.362	0.550	*d =* 0.166
SVS-S T0: M ± DS	26.77 ± 7.03	25.56 ± 6.17	F_1;51_ = 0.447	0.507	*d =* −0.183
PANAS-N T1: M ± DS	15.15 ± 6.34	13.48 ± 4.85	F_1;50_ = 0.018	0.893	*d_ppc2_ =* −0.198
PANAS-P T1: M ± DS	28.62 ± 8.70	24.70 ± 7.41	F_1;50_ = 9.501	**0.003**	*d_ppc2_ =* 0.805
SVS-S T1: M ± DS	27.42 ± 8.05	23.15 ± 7.48	F_1;50_ = 4.722	**0.035**	*d_ppc2_ =* 0.456

M, mean; N, number; SD, standard deviation; BSI, brief symptoms inventory; GSI, global severity index; PANAS-N, positive and negative affect schedule-negative affect; PANAS-P, positive and negative affect schedule-positive affect; SVS-S, subjective vitality scales-state version; T0, pre-stimuli assessment; and T1, post-stimuli assessment.

Means and SDs for T1 are not adjusted for the covariates. In bold significant values.

### Functional connectivity results

The effect sizes for *T*-threshold were 1.428, 3.571, and 5.713, corresponding, respectively, to small, medium, and large effect sizes. At T0, the thresholds for significance (corrected for multiple testing) were *T* = ±3.462 corresponding to *p* < 0.05, and *T* = ±3.883, corresponding to *p* < 0.01. In this condition, no significant differences were observed between groups.

**Figure 1:**
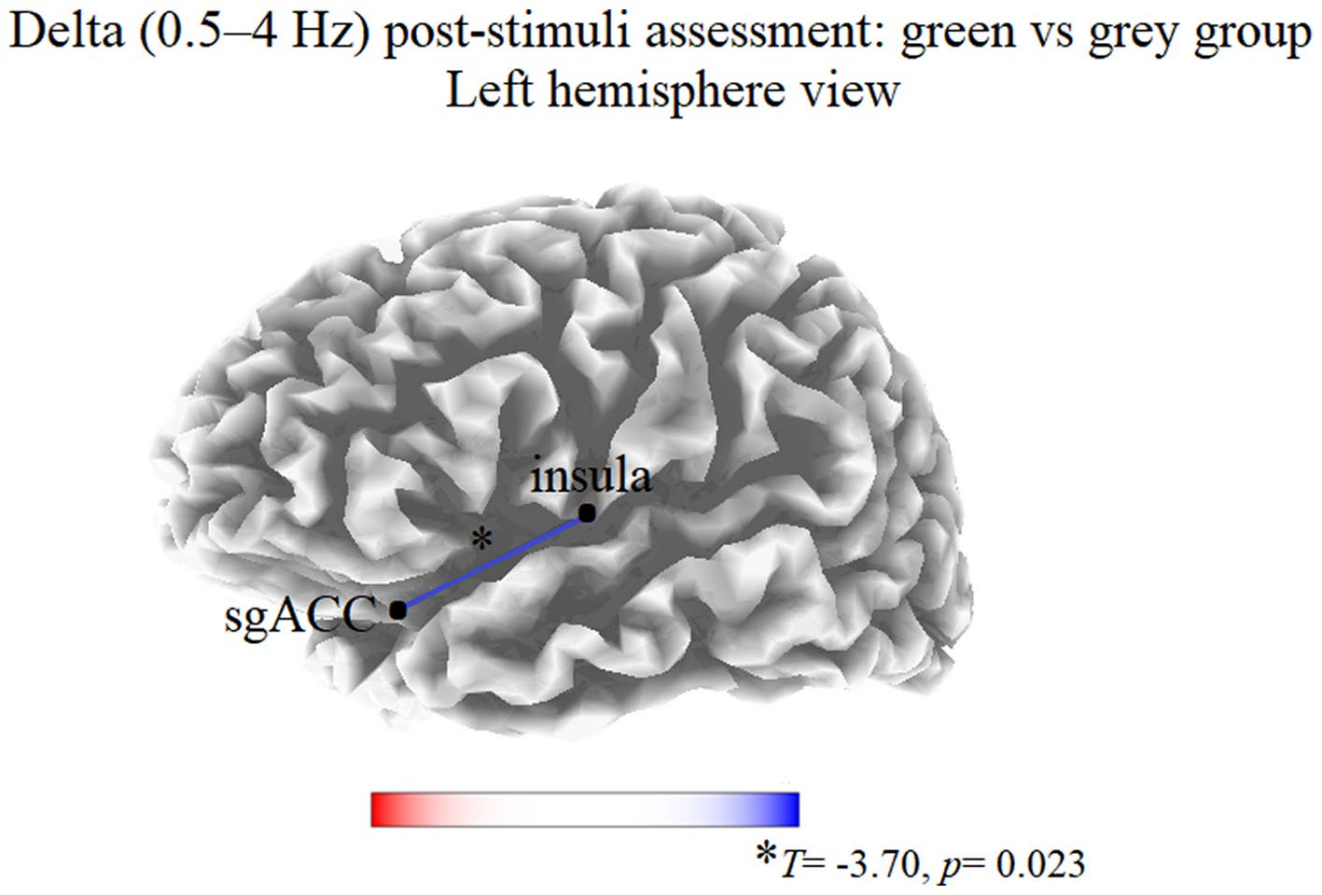
Results of the eLORETA functional connectivity between group comparison (green vs. gray) in the delta frequency band at T1. Blue lines indicate connections presenting a decrease of EEG functional connectivity. Red lines (not present) would indicate an increase of EEG functional connectivity. Threshold values (*T*) for statistical significance are reported at the bottom of the figure. sgACC, subgenual anterior cingulate cortex.

At T1, the thresholds for significance (corrected for multiple testing) were *T* = ±3.463 corresponding to *p* < 0.05, and *T* = ±3.969 corresponding to *p* < 0.01. In this condition, significant modifications were observed in delta band. Compared to the gray group, participants in the green group showed a decreased delta LPS between the left insula and left subgenual anterior cingulate cortex (sgACC; *T* = −3.70, *p* = 0.023; Figure 1). No significant differences were observed in the other frequency bands.

## Discussion

The main aim of the present study was to investigate the association between the exposure to nature and EEG functional connectivity in the distress network. Our results showed that the exposure to natural images, compared to urban pictures, was associated with a decrease of delta functional connectivity in the distress network, specifically between the left insula and left sgACC. Moreover, as compared to gray stimuli, the exposure to green spaces was also associated with a significant increase of positive emotions and the subjective experience of being full of energy and alive.

Our results would seem to be in accordance with previous neurophysiological studies suggesting that experiencing natural environments is associated with brain functional dynamics linked to emotional restorative processes and stress reduction (Grassini et al., 2019, 2022; Norwood et al., 2019; Sahni and Kumar, 2020). Indeed, both the insula and the sgACC are known to be brain regions commonly involved in emotional distress (Li et al., 2018; Pattyn et al., 2018). More specifically, while the insula plays a crucial for the integration of interoceptive information and global-emotional processing (e.g., emotional salience detection) and awareness (Duerden et al., 2013), the sgACC, given its connections with subcortical and cortical structures, is critically involved in regulating visceral and autonomic responses to stressful events, assigning emotional valence to internal and external stimuli, and emotional expression (Pizzagalli, 2011; Rolls, 2019). These two brain areas are strongly and reciprocally connected (Simmons et al., 2013) and abnormal increased resting state insula-sgACC connectivity has been suggested as a neurophysiological pattern associated to negative emotions in response to stress (Shao et al., 2018). Consistently, it has been reported that adaptive response to stress (i.e., high level of resilience) was associated with reduced sgACC-insula RS connectivity immediately following stress induction (Shao et al., 2018).

The involvement of these structures has been also documented by several studies measuring the effect of environment on brain activity. For example, the decrease of subgenual prefrontal cortex activity has been detected (Bratman et al., 2015) following the exposure to a brief nature experience (i.e., a 90-min walk in a natural setting). It has also been shown that the limbic and paralimbic areas (including the insula) were more active in uncomfortable urban environments reflecting more negative emotional stimulation (Kim and Jeong, 2014). Furthermore, previous EEG studies showed that unpleasant pictures elicited increased delta coherence over long-range circuits of the brain (Klados et al., 2009; Guntekin et al., 2017).

Intriguingly, the decrease of delta connectivity between the insula and the sgACC was detected in the left hemisphere. Although the right hemisphere dominance in emotional processing is generally recognized, the traditional division of cognitive functions according to the left–right brain dichotomy seems to have become obsolete (Stankovic, 2021). Moreover, the modern view of brain supports a dynamic large-scale brain networks model underlying different components of emotional experience (i.e., generation, perception, and regulation; Morawetz et al., 2020; Palomero-Gallagher and Amunts, 2022). For example, it has been proposed (Stankovic, 2021) that the right-biased lateralization of emotional experience can be changed rapidly due to strong environmental input such as acute stress.

Thus, considering the above mentioned findings, the current neurophysiological pattern (i.e., decreased delta coherence between the left insula and the left sgACC) might reflect the neural underpinning of the processes related to emotional restorative effects of interacting with nature. Obviously, the other side of the coin could be considered, and both are not mutually exclusive: urban scenarios can induce brain responses associated with negative emotion and stress that may be restored through the exposure to natural environments (Ancora et al., 2022).

Of relevance, no significant associations were detected in other frequency bands (e.g., alpha and theta) previously associated with nature exposure and stress reduction (Ulrich, 1981; Chang et al., 2008; Grassini et al., 2019, 2022; Sahni and Kumar, 2020; Koivisto et al., 2022). The discrepancies between previous reports and the current research may be explained by several differences in study designs and methods. More specifically, our study differs from these previous studies by investigating, for the first time, EEG functional connectivity in a pre-defined brain network (i.e., the distress network). Indeed, while EEG power reflects the amount of activity in certain frequency bands (Dressler et al., 2004), EEG connectivity provides “*information on the degree of synchrony of brain activity at different locations for each frequency, independent of power*” (Bowyer, 2016). Furthermore, compared to previous studies that evaluated the modifications of EEG parameters during the view of nature-related stimuli, we have investigated EEG data during the RS condition, which is thought to reflect the neurophysiological base rhythm providing potentially valuable information on the functional dynamic interactions between brain structures (Raichle, 2011). Thus, the current findings should be considered specific to the eyes-closed RS condition and future EEG connectivity investigations should be performed also during different task-related conditions.

The present findings should be considered while taking into account several limitations. First, our study provides an image that is informative of the dynamics that take place *right after* exposure to nature, while in future research it could be interesting to investigate if the changes observed in the neural correlates would last and, if so, for how long. These findings could also contribute to designing interventions aimed at restoration through nature exposure, providing protocols for the duration of the intervention (e.g., a certain amount of time every day for several days/weeks) and testing the short-and long-term consequences. This issue have been previously investigated by a handful of studies (e.g., Barton and Pretty, 2010; Shanahan et al., 2016), and further research is needed. Secondly, although an *a priori* power analysis was performed according to a previous EEG coherence study, the sample size was adequate for detect a large effect size. Moreover, notwithstanding university undergraduate students represent an easily accessible population, a potential source of bias should be considered (Medin et al., 2017).

Thirdly, we employed two photo slideshows showing green vs. gray spaces and found significant results in line with previous studies. Nevertheless, it would be interesting to test the replicability of the findings in outdoor nature including blue or white spaces, to see if there would be any differences in the distress network activity observed. Moreover, future studies investigating differences in neurophysiological underpinnings between the exposure to urban green spaces vs. wild green spaces are needed. Furthermore, other stress-related psychophysiological markers (e.g., the cortisol levels) in relation to nature exposure were not evaluated, and future studies would need to fill in such a gap. Lastly, while the eLORETA software is a reliable tool to investigate brain connectivity, it has an intrinsic limit in space resolution. Moreover, although 31 electrodes might be appropriate for investigating brain networks, it is known that the spatial localization accuracy of EEG increases with high-density (e.g., 256 electrodes) recording approaches (Dattola et al., 2020). Thus, our results should be treated with caution and should be deeply investigated in further studies combining multimodal neuroimaging data (i.e., high-density EEG/fMRI) in larger representative samples in order to better understand the neural dynamics underlying the restorative effect of nature.

Despite these limitations, we believe that our results have relevant theoretical and practical implications. From a theoretical point of view, these findings add up to all previous research that suggests psychophysiological restoration induced by nature exposure (Berto, 2014). Importantly, they are in line with both SRT and ART, and the integrative framework between these two theories (Ulrich, 1981; Kaplan and Kaplan, 1989; Ulrich et al., 1991; Kaplan, 1995). Indeed, the decreased delta coherence we found between the left insula and the left sgACC is consistent with the SRT postulating that nature exposure reduces physiological stress. Moreover, this neurophysiological pattern suggests decreased effort in voluntary attention as theorized in the ART.

Starting from these findings, it would be interesting to investigate the individual differences that are related to different brain dynamics in the distress network, when exposing people to nature, such as environmental identity, connectedness to nature, sensation seeking, venturesomeness, and preferences for a specific natural environment. In this regard, future studies could examine the psychophysiological dynamics in response to different types of natural environments (e.g., sea, lake, desert, mountain, and arctic) or different types of green space. In this regard, a recent systematic review (Beute et al., 2020) detected a general beneficial relation between several green space types (e.g., park, garden, and forest) and mental health, suggesting the need of further investigations focused on underlying pathways of the association between specific green spaces (e.g., types and characteristics) and individual well-being. For example a recent multimodal EEG and functional near-infrared spectroscopy study (Olszewska-Guizzo et al., 2022a), showed that the passive exposure to a “therapeutic garden” (compared to a “residential green space” and to a “busy downtown”) was associated with an improvement of mood status and a modification of brain activity (e.g., it had a moderating effect on frontal alpha asymmetry values) in both healthy and depressed individuals.

Furthermore, it could be relevant to study how the human-nature interaction might provide restoration by observing the distress network activity (e.g., hiking, gardening, and swimming in a natural area; Bratman et al., 2019). Lastly, in recent years growing attention was directed to multisensorial experiences (i.e., indoor nature experience in which the visual stimulus is teamed with auditory and/or olfactory stimuli) and virtual reality experiences (i.e., nature exposure through head-mounted displays that can guarantee a high level of immersion to the user). Thus, observing the distress network activity also in multisensorial and virtual reality nature experiences could contribute to extend this new and expanding line of research.

As mentioned before, if future studies will confirm our results, they might be relevant to build up applied psychological interventions across different fields. However, importantly, the research should first test the duration of such beneficial effects. For instance, in clinical populations (e.g., with mental disorders or general medical patients), the exposure to nature could be important in alleviating both psychological and physical symptomatology (Collado et al., 2017). Occupational psychologists might use such interventions in order to restore employees’ cognitive resources after exposure to intensive workload in the workplace. In the general population, the exposure to nature might be employed in individuals undergoing periods characterized by high levels of stress and/or negative mood. In all these cases, the research focusing on the distress network activity could offer additional insights into the dynamics involved in the restoration and help to tailor better-suited interventions for all circumstances.

## Data availability statement

The raw data supporting the conclusions of this article will be made available by the authors, without undue reservation.

## Ethics statement

The studies involving human participants were reviewed and approved by European University’s ethics review board (Prot. N. 11/2021). The patients/participants provided their written informed consent to participate in this study.

## Author contributions

CI: study design, formal analysis, data interpretation, supervision, and writing—original draft. CM, EDR, and GAC: data acquisition, formal analysis, data interpretation, and writing—review and editing. AT and LR: data acquisition, resources, and writing—review and editing. MS: resources, supervision, and writing—review and editing. CDG: data interpretation, supervision, and writing—review and editing. GA: data acquisition, data interpretation, and writing—review and editing. GC: project administration, funding acquisition, and writing—review and editing. AP: conceptualization, study design, funding acquisition, project administration, supervision, and writing—original draft. All authors contributed to the article and approved the submitted version.

## Funding

This work was part of the project named “Establishing Urban FORest based solutions in Changing Cities (EUFORICC)” financially supported by the Ministry of Education, University and Research (MIUR) of Italy (PRIN 20173RRN2S).

## Conflict of interest

The authors declare that the research was conducted in the absence of any commercial or financial relationships that could be construed as a potential conflict of interest.

## Publisher’s note

All claims expressed in this article are solely those of the authors and do not necessarily represent those of their affiliated organizations, or those of the publisher, the editors and the reviewers. Any product that may be evaluated in this article, or claim that may be made by its manufacturer, is not guaranteed or endorsed by the publisher.

## Footnotes

^1^ https://www.randomizer.org/
